# Impact of integrated preventive and curative health package on nutritional status of children under 2 years of age in the health area of Tama, Tahoua region (Niger)

**DOI:** 10.3389/fnut.2023.1259706

**Published:** 2023-10-23

**Authors:** Roberto Pedrero-Tomé, María Dolores Marrodán, Noemí López-Ejeda, Montserrat Escruela, Merce Rocaspana, Antonio Vargas, Cristian Casademont, Rui Gutiérrez, Candelaria Lanusse

**Affiliations:** ^1^Research Group in Nutritional Epidemiology (EPINUT), Unit of Physical Anthropology, Department of Biodiversity, Ecology, and Evolution, Faculty of Biological Sciences, Complutense University of Madrid, Madrid, Spain; ^2^Médecins Sans Frontières, Barcelona, Spain; ^3^Department of Nutrition and Health, Action Against Hunger, Madrid, Spain

**Keywords:** growth, anthropometric failure, wasting, stunting, small quantity lipid-based nutrient supplement

## Abstract

**Background:**

Niger, relevant in light of current political coup, is one of the countries with the worst human development indicators, characterized by high fertility rates and extremely high infant mortality rates. Food insecurity in the region is alarming, leading to high malnutrition rates in children. This study aimed to evaluate an integral preventive-curative health program targeting children aged under 2 years in the health area of Tama, district of Bouza, Tahoua.

**Methodology:**

Anthropometric follow-up data of 6,962 children aged under 2 years were included in this study. These children received complete vaccination and malaria chemoprevention, and those older than 6 months received nutritional supplementation with a small quantity of lipid-based nutrient supplements. Fundamental growth indicators (height-for-age, weight-for-height, weight-for-age, and middle-upper arm circumference) and the Composite Index of Anthropometric Failure were calculated at the beginning and end of the program (mean time spent in the program: 14.5 ± 6.6 months) The evolution of these indicators was compared with those of a sample from a vertical vaccination program conducted in the neighboring region of Madarounfa on similar dates.

**Results:**

The proportion of children without anthropometric failure decreased from 59.5 to 40.2% (*p* < 0.001), with the categories that included stunting increasing the most. When analyzing the anthropometric indicators according to the months of compliance with the program, there was a slight improvement in the indicators of acute malnutrition, whereas those of chronic malnutrition worsened significantly. However, when compared with the Madarounfa sample, the children in the present study registered a significantly lower worsening in all three indicators: height-age (−0.46 vs. -2.44; *p* < 0.001), weight-height (+0.31 vs. -0.55; p < 0.001) and weight-age (−0.03 vs. -1.63; *p* < 0.001) difference.

**Conclusion:**

The comprehensive preventive-curative health program slightly slows the worsening of cumulative malnutrition in the early years of life in complex contexts, such as southern Niger.

## 1. Introduction

The Republic of Niger is a continental country in West Africa, with three northern quarters in the Sahara and Sahel. Desert conditions in this significant part of the territory limit the development of subsistence agriculture. Rainfall varies from region to region; however, in general, rainy periods are short and unpredictable, alternating with periods of drought. These circumstances mean that the population is permanently food insecure. Between 1996 and 2019, Niger was ranked last on the Human Development Index as the poorest country in the world. In 2021 the Human Development Index was 0.400, surpassing only Chad and the Central African Republic (1).

In the same year, the synthetic fertility index (average number of children per woman) was 6.2, and the birth rate was 45.6 births per 1,000 inhabitants. Although the mortality rate was also high (14.83‰), the country has experienced a significant demographic increase, doubling its population between 2000 (10.1 million inhabitants) and 2020 (21.4 million) (2). The life expectancy was 62.4 years, almost half its inhabitants (48.5%) were aged under 14 years, and only 16.4% lived in urban areas (3, 4).

The food security situation in Niger is alarming and mainly affects the infant population, contributing to a high rate of under-five child mortality, with 115.2 deaths per 1,000 live births (5). Exclusive breastfeeding up to 6 months reaches only 23% of children, only 6% of children between 6 and 23 months have a minimum acceptable diet, and anemia is estimated to affect 73% of children under 5 years (6). Moreover, the Nutritional and Mortality Survey published by the National Institute of Statistics and elaborated according to the Standardized Monitoring and Assessment of Relief and Transitions (SMART) methodology in 2021 (7) reported prevalence rates of 12.7, 2.7, and 43.5% for global, severe acute, and chronic malnutrition, respectively. This last figure is higher than the average for the African region (30.7%) and is categorized at an emergency level, according to the criteria of the World Health Organization (WHO) (8). For this reason, the fight against malnutrition has been a priority for several years for the Nigerian government, which has been implementing a National Nutrition Security Policy for almost a decade (7).

Doctors Without Borders (Médecins Sans Frontières, MSF) have been working in Niger permanently since 2001 and have strengthened their presence there since 2005. In response to all sorts of medical challenges, particularly measles or meningitis epidemics, during a vaccination campaign in 2001, there was a high prevalence of acute malnutrition in the Maradi region, and consequently, nutritional projects were initiated to address this problem (9, 10). In addition to expanding its capacity, it sought the intervention of other Non-Governmental Organizations, and other MSF sections came in to cover other regions of the country. Furthermore, when MSF began intervening in the Tahoua region, Community Management of Acute Malnutrition (CMAM) programs using ready-to-use therapeutic foods were established.

Approximately 60,000 severely malnourished children in Niger were treated by MSF in that year (10). As a result, MSF has shifted its understanding of the malnutrition problem, realizing that a sole focus on treatment was unsuitable. Therefore, an integrated preventive and curative healthcare package, known as the PPCSI (an acronym for “Paquet préventif et curative de soins intégrés” in French), was developed with the primary aim of decreasing mortality in children aged under 5 years in a way that, if proven effective, could be replicated in more areas of the country. This package is intended to prevent and treat malnutrition, malaria, and other common diseases. Additionally, it ensures and supports vaccination and breastfeeding (11).

Stunting is a height-for-age (HAZ) score below −2 z-score from the median of the WHO growth standard (12). A longitudinal growth retardation occurs in response to cumulative nutritional deficits. This anthropometric failure reached an all-time high in Niger in 2018 (47.8%), with Tahoua being the most affected region (42.9%). Being underweight is a determinant predictor of stunting, as highlighted in different populations in Asia and sub-Saharan Africa (13–15). Moreover, insufficient micronutrients, especially zinc, iron, calcium, and vitamin A, significantly affect longitudinal growth, which is deficient after weaning in environments of low dietary diversity, such as Niger (16, 17). Unlike acute malnutrition, which can be reversed quickly by nutritional, medical, and psycho-stimulation treatments, a meta-analysis by Goudet et al. (18) reported that nutritional interventions alone did not reduce growth retardation treating chronic malnutrition that requires a multisectoral approach over a relatively long period.

Furthermore, some studies have shown modest improvements in stunting following a lipid-enriched or flour-enriched therapeutic feed (18, 19). This effect is more positive when supplementation is accompanied by vaccination and nutritional education for mothers or caregivers (20). The present study aimed to analyze the effect of a PPCSI, which included a small quantity of lipid-based nutrient supplement (SQ-LNS) as a food complement, on the nutritional status of children aged between 6 and 24 months in Tahoua, Niger.

## 2. Materials and methods

### 2.1. Study design

**Figure 1:**
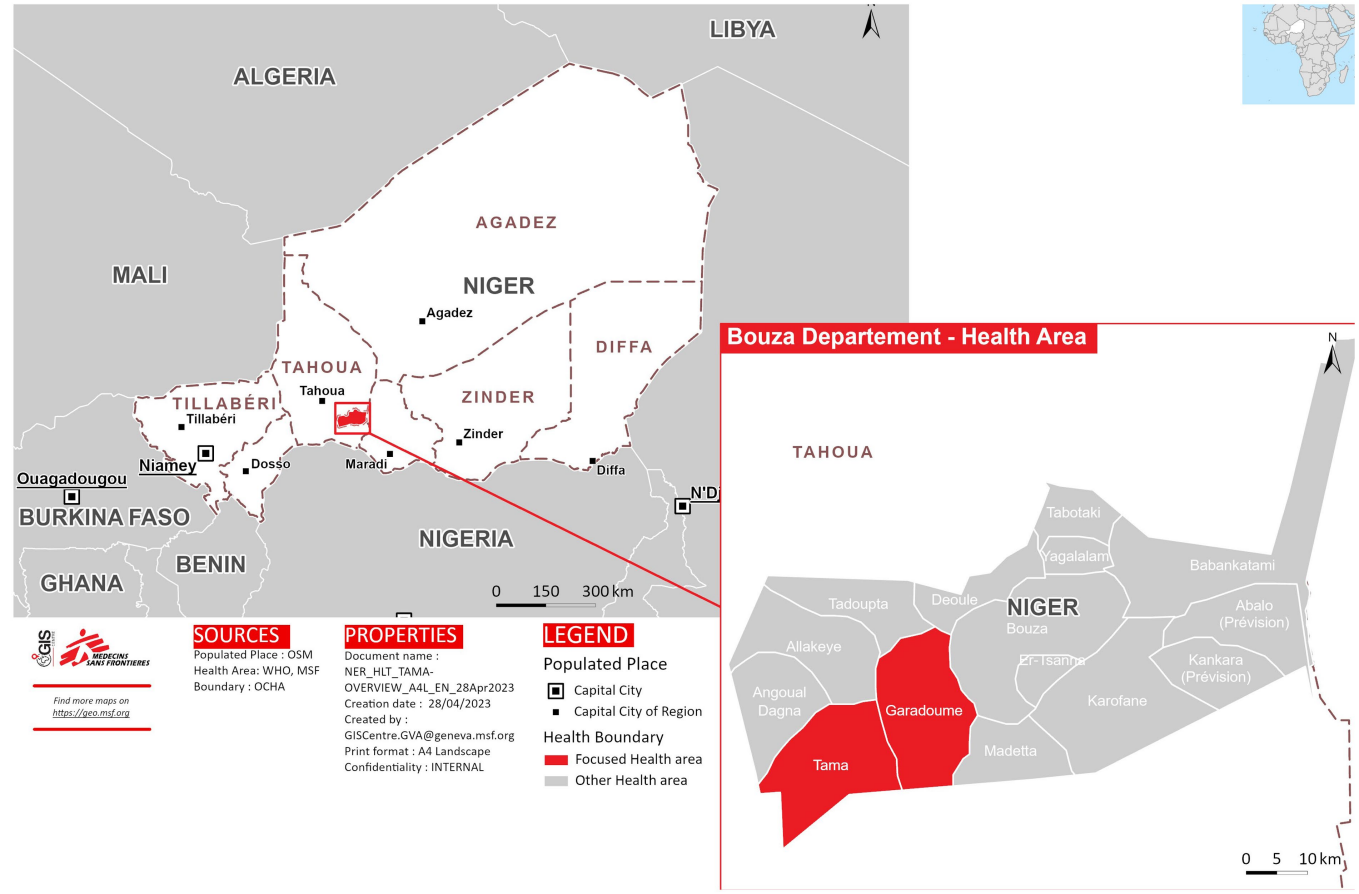
Geographic location of the health areas involved in the study (own source).

The PPCSI was conceived as an operational research project with a prospective cohort methodology implemented by MSF in the Tama health area, located in the Bouza health district of the Tahoua region (Figure 1), where MSF conducted a humanitarian assistance project in agreement with the Ministry of Health of Niger. The study was also approved by the National Ethics Committee in Niger (Comité Consultatif National d’Ethique, reference 013/2014/CCNE, 2014) and the MSF Ethics Review Board (ID 1535, 2015), and compliance with the country’s current health legislation was ensured.

The intervention targeted all children under 24 months of age and residing in the Tama health area during the 3 years and 9 months of the PPSCI (March 2015–December 2018), and during that time, 8,116 children aged between 0 and 23 months were admitted. This sample was recruited through all health posts located in Tama with the help of community health workers. All children under 2 years of age were invited to participate, and the exclusion criterion was that they presented acute malnutrition at the beginning since they were referred to another specific treatment program. Written informed consent of parents or guardians was obtained after explanations in the local language, guaranteeing that refusal to participate would not affect access to free care in MSF-supported facilities. On admission, the children received seasonal malaria chemoprevention and complete vaccination (polio, tuberculosis, pentavalent rotavirus, pneumonia, yellow fever, and measles). Additionally, for those aged over 6 months, deworming treatment every 6 months and supplementary feeding with one Nutributter© sachet daily (20 g, 108 kcal) were provided. This daily ration contained 2.6 g of protein and 7 g of fat (essential fatty acids) as well as vitamins A (0.4 mg), B1 (0.3 mg), B2 (0.4 μg), B3 (1.8 mg), B6 (0.3 mg) B12 (0.5 mg) C (30 mg) and folic acid (80 μg). Moreover, it incorporated minerals such as calcium (100 mg), potassium (152 mg), zinc (4 mg), iron (9 mg), selenium (10 mg), phosphorus (82 mg), magnesium (16 mg), copper (0.2 mg), iodine (90 mg) and manganese (0.08 mg) (21). At the same time, the mothers received education on breastfeeding and infant nutrition. The conveyed message was mainly about basic hygiene and the importance of breastfeeding. Giving babies under 6 months only breast milk, without other liquids such as water or tea, was recommended, a common habit in the country’s cultural practices. This program has been previously described in detail (11). It should be noted that the investigators have no record of whether the sachets with the food supplement were ingested in their entirety by the child. This is an important aspect that, unfortunately, could not be controlled.

Monthly medical follow-ups (visits) were established. to check the children’s health and growth; measure weight, length, or height; and middle-upper arm circumference (MUAC) to make an anthropometric diagnosis of their nutritional condition. Anthropometric measurements were performed by MSF-trained health personnel using salter-type scales (100 g accuracy), baby/infant length/height wooden measuring boards, and standard MUAC tapes. However, inter- and intra-observer controls were not performed to ensure an acceptable technical measurement error. This circumstance may perhaps explain the number of implausible observations that had to be eliminated from the database.

### 2.2. Data processing

**Figure 2:**
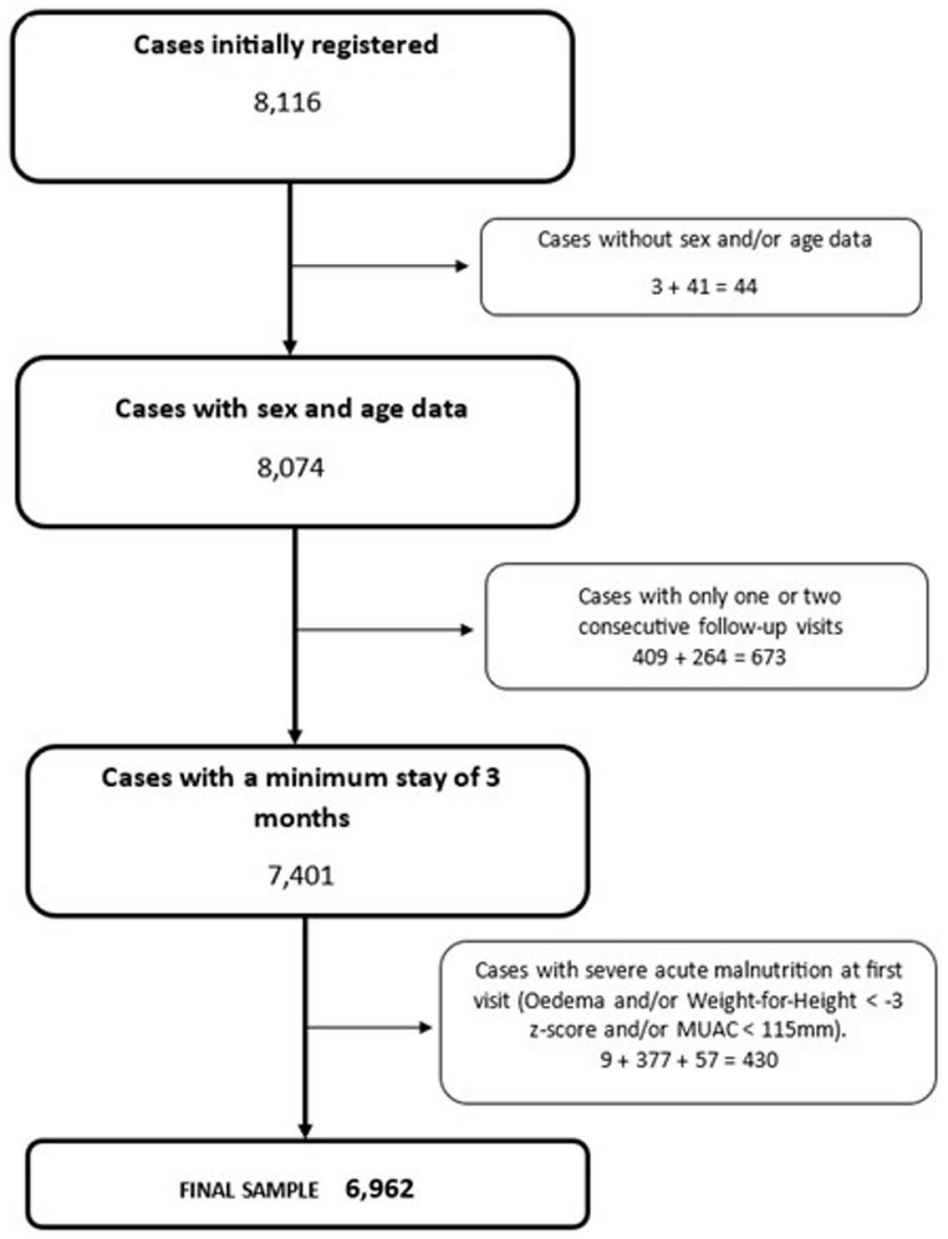
A flowchart depicting the database cleaning process.

Figure 2 shows the workflow used to clean the database. Firstly, data of all participants whose sex or age was not recorded were discarded, as these were considered critical variables in the analysis to establish nutritional status. Secondly, we eliminated participants who made fewer than three visits, which is the minimum time estimated to be able to appreciate the changes associated with the intervention. Finally, participants diagnosed with severe acute malnutrition (SAM) at their first visit were excluded because they were referred to CMAM treatment programs. Data from 14.4% of the initially registered participants (N = 8,116) were discarded, and the final database included 6,962 children under 24 months who attended a health post in the Tama health area.

Table 1 summarizes the results of the second data-cleaning process. In this case, the exclusion criterion does not correspond to the eliminated individuals but to anomalous data, that is, values incompatible with life according to WHO fixed exclusion criteria (22). WHO Antro software (23) was used to estimate the z-scores for the HAZ, weight-for-height (WHZ), and weight-for-age (WAZ) indicators that evaluate chronic, acute, and global malnutrition, respectively. Thus, data with HAZ values below −6 z-score or above +6 z-score, WHZ values below −6 z-score or above +5 z-score, and WAZ values below −5 z-score or above +5 z-score were eliminated. As can be seen from the amount of data eliminated, the error rate was higher in measuring body length indicators compared to body weight.

**Table 1:** Anthropometric records eliminated by indicator and follow-up visits.

	Height-for-age	Weight-for-height	Weight-for-age	Total by visit
Initial data	89,335	89,397	91,891	27,0623
Visit 1	295	481	75	851
Visit 2	316	397	40	753
Visit 3	358	479	42	879
Visit 4	35	466	42	543
Visit 5	335	372	25	732
Visit 6	270	256	30	556
Visit 7	231	153	30	414
Visit 8	194	117	22	333
Visit 9	161	82	18	261
Visit 10	118	53	17	188
Visit 11	92	34	20	146
Visit 12	60	22	18	100
Visit 13	41	16	11	68
Visit 14	29	8	10	47
Visit 15	11	6	8	25
Visit 16	11	4	3	18
Visit 17	8	4	6	18
Visit 18	10	5	6	21
Visit 19	5	3	4	12
Visit 20	0	1	1	2
Visit 21	1	1	1	3
Visit 22	0	0	0	0
Visit 23	0	0	0	0
Visit 24	1	1	0	2
Total data eliminated	2.89% (2,582)	3.31% (2,961)	0.47% (429)	2.21% (5,972)

The final sample consisted of 6,962 children (3,559 boys and 3,403 girls) aged between 0 and 23 months. The mean age of entry into the nutritional program was 2.8 (inter-quartile range 1.2–9.0) months. Of the children, 65.1% started the program before 6 months of age, 17.4% between 6 and 11 months of age, 11.8% between 12 and 17 months of age, and 5.7% between 18 and 24 months of age. The mean time in the program was 14.5 ± 6.6 months. Notably, 15.2% of the children did not continue the program until a period of 6 months and 36.0% exceeded 18 months in the program.

### 2.3. Statistical analysis

To contrast the possible effect of the length of stay in the program, quartiles were established for this variable, and the Wilcoxon test was applied to compare the averages corresponding to the z-scores of the different anthropometric indicators (HAZ, WHZ, and WAZ) and MUAC. In contrast, the Composite Index of Anthropometric Failure (CIAF) (24) was estimated and compared for each category and the average z-scores of the different anthropometric indicators at the beginning and end of the program.

Stunting, wasting, and underweight reflect distinct biological processes, but the same subject may exhibit more than one of these characteristics simultaneously. At the population level, CIAF is a measure that provides a single, aggregated figure for the number of children affected. This index identifies six groups in which category A encompasses children without anthropometric failure. The other five are B: wasting only; C: wasting and underweight; D: wasting, stunting, and underweight; E: stunting and underweight; F: stunting only; and Y: underweight only. The CIAF is calculated by subtracting those in group A from the total number of children in the sample.

As a comparison or proxy control group, we used the database compiled by the MSF in the work of Kohlmann et al. (25) to explore the association between chronic and acute malnutrition and its ontogenetic evolution in children under 2 years. This database grouped all individuals analyzed in a previous case–control study (26) to test the effectiveness of an oral vaccine against rotavirus, which causes gastroenteritis. That study was conducted in the health district of Madarounfa in the Maradi region (bordering the region of the present study) on nearby dates (August 2014 to December 2019). Additionally, the sample size (N = 6,567), age range (between 6 weeks and 24 months), and periodicity of visits for monitoring growth (every 4 weeks; 139,529 visits in total) were similar to those of the present study. Therefore, the Madarounfa database was considered adequate as a control series, as the children did not receive any nutritional supplementation.

Violin plots were used to establish the contrast in the progression of nutritional status over time, showing the differences in the HAZ, WHZ, and WAZ z-scores between the first and last visits and comparing the results obtained in the present study with those achieved in the Madarounfa region. All the statistical analyzes were performed using R software (v.4.3.1).

## 3. Results

Table 2 shows the anthropometric indicators at the beginning and end of the program for all children who completed a minimum of three visits. The z-scores for the HAZ, WHZ, and WAZ moved away from the reference toward malnutrition values at the end of the intervention. In contrast, the MUAC increased by an average of 0.5 cm. When the CIAF was analyzed, the anthropometric failure increased by approximately 20%. This increase in malnutrition was best observed in the nutritional categories that included stunting.

**Table 2:** Anthropometric indicators at the beginning and end of the follow-up for the whole sample*.

Indicator	*N*	Time point	Median [IQR]	Value of *p*
Height-for-age (z-score)	8,040	START	−1.30 [−2.40, −0.25]	<0.001
		END	−2.01 [−2.94, −1.11]	
Weight-for-height (z-score)	7,672	START	−0.28 [−1.12, 0.89]	<0.001
		END	−0.40 [−1.21, 0.42]	
Weight-for-age (z-score)	7,630	START	−0.92 [−1.79, −0.12]	<0.001
		END	−1.36 [−2.1, −0.65]	
MUAC (mm)	7,153	START	135 [130, 140]	<0.001
		END	140 [135, 148]	

CIAF category	Time point	% (*N*)	Value of *p*
Without anthropometric failure	START	86.2% (5,606)	<0.001
	END	28.9% (1,877)	
Wasting only	START	4.3% (277)	
	END	0.3% (21)	
Wasting + Underweight	START	1.8% (118)	
	END	2.6% (170)	
Stunting + wasting + underweight	START	0.6% (37)	
	END	9.3% (605)	
Stunting + underweight	START	2.6% (170)	
	END	27.7% (1,801)	
Stunting only	START	3.3% (215)	
	END	29.8% (1,937)	
Underweight only	START	1.2% (77)	
	END	1.4% (89)	

CIAF, Composite Index of Anthropometric Failure; IQR, interquartile range; MUAC, middle-upper arm circumference; * Includes girls and boys who entered the program at different ages. Mean time spent in the program: 14.5 ± 6.6 months.

Table 3 shows the anthropometric results at the beginning and end of the follow-up period, separating children who complied with the complete program from those who did not. Those who had not completed the program for 24 months were children who were not enrolled at birth and, therefore, entered the program at an older age, SAM cases referred for CMAM treatment programs, and dropouts before 24 months. Table 4 shows the anthropometric changes according to the length of stay in the program. The acute malnutrition indicator improved significantly, whereas the growth retardation and underweight indicators worsened.

**Table 3:** Anthropometric indicators at the beginning and end of the follow-up according to compliance with the whole program.

Children who completed the whole program (from 0 to 24 months)				
Indicator	*N*	Time point	Median [IQR]	Value of *p*
Height-for-age (z-score)	1,566	START	−1.20 [−2.30, −0.15]	<0.001
		END	−1.93 [−2.86, −1.00]	
Weight-for-height (z-score)	1,542	START	−0.22 [−1.22, 0.98]	0.068^NS^
		END	−0.41 [−1.24, 0.44]	
Weight-for-age (z-score)	1,542	START	−0.85 [−1.72, −0.07]	<0.001
		END	−1.32 [−2.10, −0.58]	

Children who did not complete the whole program				
Indicator	*N*	Time point	Median [IQR]	Value of *p*
Height-for-age (z-score)	6,474	START	−1.69 [−2.72, −0.68]	<0.001
		END	−2.40 [−3.16, −1.53]	
Weight-for-height (z-score)	6,130	START	−0.51 [−1.23, −0.61]	<0.001
		END	−0.35 [−1.10, 0.34]	
Weight-for-age (z-score)	6,088	START	−1.17 [−2.03, −0.36]	<0.001
		END	−1.49 [−2.16, −0.89]	

IQR, interquartile range; NS, not significant.

**Table 4:** Anthropometric indicators at the beginning and end of the follow-up according to the months of participation in the program.

Children with length of stay within the first quartile (< 9 months)				
Indicator	*N*	Time point	Median [IQR]	Value of *p*
Height-for-age (z-score)	1,058	START	−1.91 [−2.92, −0.94]	<0.001
		END	−2.31 [−3.18, −1.36]	
Weight-for-height (z-score)	1,066	START	−0.77 [−1.37, −0.05]	<0.001
		END	−0.50 [−1.29, 0.24]	
Weight-for-age (z-score)	1,070	START	−1.53 [−2.24, −0.66]	<0.001
		END	−1.58 [−2.41, −0.80]	

Children with length of stay within the second quartile (9–16 months)				
Indicator	*N*	Time point	Median [IQR]	Value of *p*
Height-for-age (z-score)	1,067	START	−1.62 [−2.42, −0.74]	<0.001
		END	−2.31 [−3.03, −1.39]	
Weight-for-height (z-score)	1,073	START	−0.67 [−1.29, 0.03]	<0.001
		END	−0.26 [−1.05, 0.58]	
Weight-for-age (z-score)	1,070	START	−1.34 [−2.02, −0.61]	<0.001
		END	−1.42 [−2.14, −0.73]	

Children with length of stay within the third quartile (16–21 months)				
Indicator	*N*	Time point	Median [IQR]	Value of *p*
Height-for-age (z-score)	1,054	START	−1.33 [−2.36, −0.27]	<0.001
		END	−2.29 [−3.08, −1.47]	
Weight-for-height (z-score)	1,056	START	0.50 [−0.86, 1.16]	<0.001
		END	−0.41 [−1.20, 0.33]	
Weight-for-age (z-score)	1,054	START	−0.85 [−1.72, −0.70]	<0.001
		END	−1.45 [−2.10, −0.85]	

Children with length of stay within the fourth quartile (>21 months)				
Indicator	*N*	Time point	Median [IQR]	Value of *p*
Height-for-age (z-score)	970	START	−1.01 [−2.18, 0.11]	<0.001
		END	−2.23 [−2.97, −1.48]	
Weight-for-height (z-score)	970	START	0.41 [−0.99, 1.61]	<0.001
		END	−0.59 [−1.30, 0.25]	
Weight-for-age (z-score)	970	START	−0.43 [−1.25, 0.32]	<0.001
		END	−1.53 [−2.22, −0.91]	

IQR, interquartile range.

Finally, we compared the z-scores according to the anthropometric failure category at the beginning of the program (Table 5). About the HAZ, children who were exclusively classified as chronically undernourished achieved a slight improvement in their z-scores. In the case of the WAZ, improvement was found in children with more than one type of malnutrition (C, D and E ICAF categories).

**Table 5:** Anthropometric indicators at the beginning and end of the follow-up according to the initial anthropometric failure status.

Height-for-age Z-score			
CIAF category at inclusion	Time point	Median [IQR]	Value of *p*
Without anthropometric failure	START	−0.48 [−1.31, 0.08]	<0.001
	END	−1.57 [−2.40, −0.78]	
Wasting only	START	1.47 [0.52, 2.20]	<0.001
	END	−1.51 [−2.51, −0.60]	
Wasting + underweight	START	−0.79 [−1.49, −0.28]	<0.001
	END	−2.20 [−3.09, −1.40]	
Stunting + wasting + underweight	START	−3.13 [−3.71, −2.39]	0.584^NS^
	END	−3.27 [−4.01, −2.50]	
Stunting + underweight	START	−3.33 [−3.84, −2.67]	<0.001
	END	−3.17 [−3.81, −2.45]	
Stunting only	START	−2.76 [−3.05, −2.25]	<0.001
	END	−2.48 [−3.17, −1.80]	
Underweight only	START	−1.64 [−3.05, −1.84]	<0.001
	END	−2.35 [−1.69, −1.48]	

Weight-for-height Z-score			
CIAF category at inclusion	Time point	Median [IQR]	Value of *p*
Without anthropometric failure	START	0.02 [−0.89, 0.78]	<0.001
	END	−0.22 [−1.04, 0.55]	
Wasting only	START	−3.04 [−3.54, −2.30]	<0.001
	END	−0.41 [−1.37, 0.35]	
Wasting + underweight	START	−3.39 [−4.06, −2.54]	<0.001
	END	−0.76 [−1.65, 0.13]	
Stunting + wasting + underweight	START	−2.74 [−3.04, −2.21]	<0.001
	END	−1.12 [−1.93, −0.22]	
Stunting + underweight	START	−0.58 [−1.34, −0.10]	0.475^NS^
	END	−0.66 [−1.46, 0.12]	
Stunting only	START	1.31 [0.07, 2.42]	<0.001
	END	−0.25 [−1.13, 0.52]	
Underweight only	START	−1.54 [−1.84, −1.32]	<0.001
	END	−0.87 [−1.55, −0.23]	

Weight-for-age Z-score			
CIAF category at inclusion	Time point	Median [IQR]	Value of *p*
Without anthropometric failure	START	−0.31 [−1.02, 0.26]	<0.001
	END	−1.06 [−1.71, −0.39]	
Wasting only	START	−0.77 [−1.44, −0.23]	<0.001
	END	−1.18 [−1.82, −0.57]	
Wasting + underweight	START	−2.73 [−3.10, −2.30]	<0.001
	END	−1.79 [−2.54, −1.06]	
Stunting + wasting + underweight	START	−3.67 [−4.19, −3.10]	<0.001
	END	−2.58 [−3.25, −1.97]	
Stunting + underweight	START	−2.68 [−3.00, −2.24]	<0.001
	END	−2.22 [−2.86, −1.63]	
Stunting only	START	−1.07 [−1.64, −0.67]	<0.001
	END	−1.54 [−2.16, −0.97]	
Underweight only	START	−2.21 [−2.29, −2.06]	<0.010
	END	−1.91 [−2.43, −1.15]	

CIAF, Composite Index of Anthropometric Failure; IQR, interquartile range; NS, not significant.

**Figure 3:**
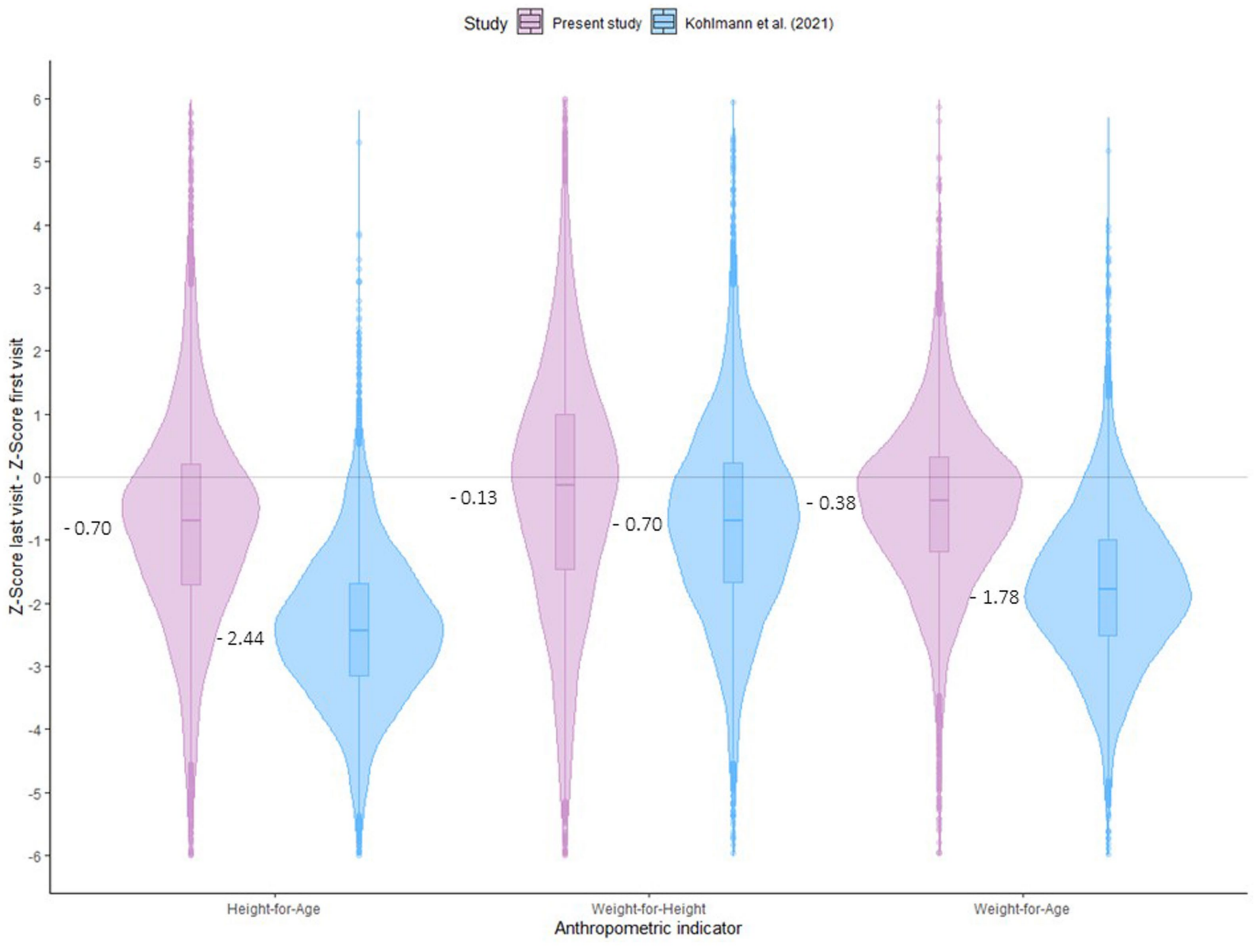
Difference between anthropometric indicators at the beginning and end of the program in two south Niger cohorts.

The contrast between the present Tahoua sample and the Madarounfa series is shown in Figure 3. As can be seen from the differences in z-scores between the first and last visits, in both series, there is a nutritional deterioration of a greater depth in the case of the HAZ. However, for all indicators considered (HAZ, WHZ, and WAZ), the difference between the first and last visits was significantly smaller in the present study compared to that from Madarounfa (*p* < 0.001).

## 4. Discussion

The CIAF increased by almost 20% over the 3 years of PPCSI in Tahoua. However, this does not invalidate its success, given that the program seems to reduce the potentially expected malnutrition burden compared with other preventive interventions in the Madarounfa border region on similar dates (25). Considering that children diagnosed with SAM at the first visit were referred for specific treatment, it can be affirmed that the PPCSI succeeded in reducing mild or moderate acute malnutrition. This occurred even among the children who only completed part of the program. Additionally, children diagnosed with stunting only showed significantly improved HAZ. Children with more than one type of malnutrition at enrollment also showed improved nutritional status, with their WHZ and WAZ approaching the median of the WHO reference (12).

During the development of PPCSI, the CIAF increased at the expense of categories that included stunting. This result was not surprising because longitudinal growth retardation accumulates with age in this context. This situation has been explained in detail by Victora et al. (27), who analyzed a large sample of studies in 54 countries worldwide. In this work, it was observed that, in the series of 30 countries in sub-Saharan Africa, the HAZ declined sharply up to 2 years of age (−0.10 z-score/month) and then increased slightly (0.005 z-score/month) up to 5 years of age.

This model was also described by Bhutta et al. (28), who reported that longitudinal growth retardation increases rapidly between 3 and 24 months of age, continues to increase more slowly until 36 months, and usually remains stable until 5 years of age. A meta-analysis by the authors above (28) analyzed the effects of more than 100 nutritional interventions conducted in 36 countries. Depending on the type of intervention (promotion of breastfeeding, supplementation with micronutrients, accompanied or not accompanied by community nutritional education, etc.), stunting was reduced by 10 to 33% at 24 months. However, the pattern (a faster increase up to 24 months and a slower increase up to 36 months) was maintained.

Food supplements are essential to nutritional recovery, and micronutrients are particularly effective in improving children’s health, survival, growth, and functional as highlighted by a review study (29). However, a more recent meta-analysis (18) provides no evidence that complementary interventions increase HAZ in poor urban settings in countries such as Bangladesh, India or Peru. As the authors rightly state, this is a wake-up call on the need to change the structural factors (social, political) that limit the effectiveness of interventions focused on the nutritional improvement of children.

In the PPCSI developed by MSF in the Tahoua region, an SQ-LNS was provided to all children aged 6 to 24 months. This home fortification adds fatty acids and energy to micronutrient and macronutrient supplementation in an easy-to-implement and acceptable manner. The daily ration provides slightly more than one-tenth of the necessary energy and practically covers the daily requirements of micronutrients in children under 2 years (30). Additionally, the product is well accepted by children and causes little digestive discomfort compared with other supplements with similar characteristics (31, 32).

Several studies have reported the benefits of supplementation with SQ-LNS in reducing anemia and improving motor development in children. However, evidence of the effect on height growth is weak. A supplementary feeding program implemented in refugee camps in Djibouti and Kenya between 2008 and 2011 with the same SQ-LNS found that among children under 5 years, anemia decreased by between 9.3 and 29.33% (depending on the age group). In contrast, the prevalence of stunting remained similar (33). Similarly, another study of 750 children aged between 6 and 12 months in the Matlosana municipality (Northwest South Africa) showed that supplementation with SQ-LNS products improved hemoglobin and iron concentrations. These supplements reduced iron-deficiency anemia but showed only a transitory effect on longitudinal growth and failed to reduce chronic malnutrition (34).

In contrast, a study conducted in Ghana on children aged 6 to 12 months, comparing groups of children receiving three nutritional products (crushable flavored multiple-micronutrient tablets, micronutrient powders, and SQ-LNS), showed that all three groups had decreased anemia and improved motor development. However, only the children supplemented with SQ-LNS had accelerated linear growth, achieving HAZ averages closer to the median of the WHO standard (HAZ = −0.20 ± 0.54) compared with those supplemented with tablets (HAZ = − 0.39 ± 0.54) or with the tablets plus micronutrient powder combination (HAZ = −0.38 ± 0.54) (35). Similarly, the results of a trial conducted on a sample of Haitian children (*n* = 589; 6–11 months) recruited from the urban slum of Haitian Cap (36) are worth mentioning. In this trial, those supplemented with the same SQ-LNS for 3 or 6 months showed more accelerated growth in height than those that did not receive supplementation. Moreover, the differences with the control group were maintained for up to 6 months after the end of the intervention. Other studies have shown additional benefits of such interventions, especially in preventing wasting (37) and reducing mortality (38). Very few studies have had such an integral approach, and none involving the SQ-LNS has been implemented in Niger, which is of high public health relevance.

The World Food Program and United Nations Children’s Fund (UNICEF) recommend distributing lipid-based supplements, such as those used in the present study, and others, such as fortified blended foods, to prevent acute malnutrition and stunting in situations of food vulnerability (39, 40). Beyond the nutritional value of a particular product, scientific evidence highlights that success in reducing anthropometric failure is more likely when multisectoral interventions are implemented, combining specific nutrition-sensitive methods and programs. Agricultural improvements, female empowerment, vaccination, parasite control, and other sanitation and hygiene measures maximize the impact of complementary or therapeutic foods (39, 40). Further research is needed to identify the most cost-effective interventions to ensure sustainability in complex contexts, such as southern Niger.

A comparison of the children analyzed in the present study with the Madarounfa series (25) highlighted the possible beneficial effect of SQ-LNS supplementation in the context of PPCSI on the anthropometric condition of the children. Both studies were carried out in Niger by the MSF on similar dates, with similar sample sizes and approaches to growth monitoring (present study: *N* = 6,962 children; visits = 92,517; Madarounfa: *N* = 6,567 children; 139,529 visits). The Madarounfa study used prospective data from a double-masked placebo-controlled trial to evaluate the efficacy and safety of a multivalent vaccine against bovine rotavirus and severe rotavirus gastroenteritis. It should be noted that some mothers received different types of prenatal supplements: lipid-based supplements, multiple micronutrient supplements, or iron-folic acid (41, 42). As shown in the results, the z-scores between the first and last visits indicated a deterioration in growth in anthropometric indicators that combine weight, height, and age. However, this impairment was more pronounced in the Madarounfa series, especially in the HAZ and WAZ.

The present results show that integral interventions, such as PPCSI, can positively impact complex contexts, contributing to increased vaccination rates, expanding seasonal malaria chemoprevention activities, screening and treating children with malnutrition and several diseases, and training and involving the community as a pillar of the intervention. The duration and integral approach of the program in complex settings with high levels of all forms of undernutrition need to catch up in its attempt to improve this situation. Nonetheless, all the efforts in this direction are relevant.

At this point, the authors believe it is essential to reflect on the scope of the interventions that specific organizations such as MSF carry out in contexts of humanitarian crisis and severe food insecurity. These interventions are based on improving the most vulnerable groups’ nutrition and primary sanitary and hygienic conditions. As has been shown in this paper and others cited, the impact of these actions partly slows down the deterioration of nutritional status, slowing down the worsening of stunting.

However, the success of these programs is strongly limited by the fact that child growth is a process that is part of a holistic Social-Economic-Political-Emotional (SEPE) process. This concept focuses on the interaction between the biology of development and the quality of material and societal conditions (43, 44).

This means that nutritional status does not depend solely on diet and controlling infections and parasitosis. All the SEPE factors, not strictly nutritional, significantly influence human growth. In the environments where humanitarian actions are implemented, conditions of inequity, low educational levels, insecurity, or violence are common, translating into chronic stress. All this affects children emotionally, undermining the production of hormones such as oxytocin and osteocalcin, which are involved in skeletal development and regulate height expression. In this regard, some authors (45, 46) discuss extensively how stress, education, socioeconomic, political, and emotional conditions are responsible for stunting.

We must remember that intake and disease are the immediate causes of malnutrition. However, the underlying and fundamental causes, such as the economic, political, and ideological structure of countries, primarily generate the damage. It is very complex to promote changes at this level. However, we must be aware that the programs promoted by NGOs can only succeed in addressing the problem from an ecological and global perspective. In order to have an average growth, children need proper nutrition and protection against violence, abuse, neglect, environmental threats, including air pollution, and prolonged exposure to other adversities that arise in countries in crisis or conflict situations (47). To achieve effectiveness, there is a need to strengthen the role of preventing malnutrition and other diseases, focusing on the whole context of the social determinants of health (48).

The main strengths of the present study are that it was conducted at the community level, with a large number of Community Health Workers performing home visits, recruiting children, and following up on them, thereby enhancing adherence to the program. However, this study had some limitations. This operational study was based on the anthropometric follow-up of a cohort of children participating in a new integrated healthcare program. Therefore, the main limitation of this study was the need for a formal control group (children from a neighboring health area where the program had not been implemented). To assess the program’s impact, data collected in another MSF operational study in a border region with similar socio-environmental conditions were used; however, this could not be objectively assessed. Consequently, the program’s impact results should be interpreted with caution.

Additionally, anthropometric follow-up was performed by the health staff of the health centers after receiving training for this purpose; however, their lack of experience may have caused measurement errors. This has resulted in eliminating several anthropometric indicators owing to their implausibility. Typing or missing information could not be prevented, and rounding up height measurements was common. Additionally, relevant information on the roles and activities of Community Health Workers was unavailable. It is still being determined whether they supervised the acceptability and consumption of SQ-LNS (except for the collection of empty supplement sachets at every visit). Furthermore, intake of other local foods was not assessed.

During data cleaning, several children who presented with SAM on admission were excluded from the analysis. However, when they returned to PPCSI after recovery from the CMAM programs, this circumstance was not recorded and could result in differential growth. A better method to follow up with children back and forth between both programs would have provided a more precise overview of the program, and an analysis of the interference between stunting and acute malnutrition could have been executed.

In conclusion, the PPCSI program, which integrates vaccination, malaria chemoprevention, identification and treatment of malnutrition and other diseases, and supplementation with SQ-LNS for all children aged under 2 years, is slightly effective in curbing the accumulated burden of malnutrition in the early years of life in complex contexts, such as that in southern Niger.

## Data availability statement

The raw data supporting the conclusions of this article will be made available by the authors, without undue reservation.

## Ethics statement

The studies involving humans were approved by National Ethics Committee in Niger (Comité Consultatif National d’Ethique, reference 013/2014/CCNE, 2014) and the MSF Ethics Review Board (ID 1535, 2015). The studies were conducted in accordance with the local legislation and institutional requirements. Written informed consent for participation in this study was provided by the participants’ legal guardians/next of kin. Written informed consent was obtained from the individual(s) for the publication of any potentially identifiable images or data included in this article.

## Author contributions

RP-T: Formal analysis, Writing – original draft, Writing – review & editing. MM: Formal analysis, Writing – original draft, Writing – review & editing. NL-E: Writing – original draft, Writing – review & editing. ME: Writing – original draft, Writing – review & editing. MR: Conceptualization, Methodology, Writing – review & editing. AV: Conceptualization, Methodology, Writing – review & editing. CC: Conceptualization, Methodology, Writing – review & editing. RG: Conceptualization, Methodology, Writing – review & editing. CL: Conceptualization, Methodology, Writing – review & editing.

## Abbreviations

MSF: Médecins Sans Frontières
CMAM: Community Management of Acute Malnutrition
PPCSI: Paquet préventif et curatif de soins intégrés
HAZ: height-for-age
WHZ: weight/height
WAZ: weight/age
WHO: World Health Organization
SQ-LNS: lipid-based nutrient supplement
UNICEF: United Nations International Children’s Emergency Fund
MUAC: middle-upper arm circumference
CIAF: Composite Index of Anthropometric Failure
SAM: severe acute malnutrition
